# A dataset describing a suite of novel antibody reagents for the RAS signaling network

**DOI:** 10.1038/s41597-019-0166-7

**Published:** 2019-08-29

**Authors:** Regine M. Schoenherr, Dongqing Huang, Uliana J. Voytovich, Richard G. Ivey, Jacob J. Kennedy, Richard G. Saul, Simona Colantonio, Rhonda R. Roberts, Joseph G. Knotts, Jan A. Kaczmarczyk, Candice Perry, Stephen M. Hewitt, William Bocik, Gordon R. Whiteley, Tara Hiltke, Emily S. Boja, Henry Rodriguez, Jeffrey R. Whiteaker, Amanda G. Paulovich

**Affiliations:** 1 Fred Hutchinson Cancer Research Center, Seattle, WA USA; 2 Cancer Research Technology Program, Antibody Characterization Lab, Frederick National Laboratory for Cancer Research, Frederick, MD USA; 3 National Cancer Institute, Bethesda, MD USA

**Keywords:** Immunoprecipitation, Mass spectrometry, Cancer, Immunohistochemistry, Immunoblotting

## Abstract

*RAS* genes are frequently mutated in cancer and have for decades eluded effective therapeutic attack. The National Cancer Institute’s RAS Initiative has a focus on understanding pathways and discovering therapies for RAS-driven cancers. Part of these efforts is the generation of novel reagents to enable the quantification of RAS network proteins. Here we present a dataset describing the development, validation (following consensus principles developed by the broader research community), and distribution of 104 monoclonal antibodies (mAbs) enabling detection of 27 phosphopeptides and 69 unmodified peptides from 20 proteins in the RAS network. The dataset characterizes the utility of the antibodies in a variety of applications, including Western blotting, immunoprecipitation, protein array, immunohistochemistry, and targeted mass spectrometry. All antibodies and characterization data are publicly available through the CPTAC Antibody Portal, Panorama Public Repository, and/or PRIDE databases. These reagents will aid researchers in discerning pathways and measuring expression changes in the RAS signaling network.

## Abstract (summary)

**Table:** 

Design Type(s)	disease analysis objective
Measurement Type(s)	immunocapture • immunoprecipitation • antibody • in situ immunoassay • ex situ immunoassay
Technology Type(s)	western blot analysis • liquid chromatography-tandem mass spectrometry • immuno-MRM • immunohistochemistry • RPPA protein profiling assay
Factor Type(s)	
Sample Characteristic(s)	Homo sapiens • MCF-10A cell • BxPC-3 cell • NCI cell • breast • ovary • colon • lung • A549 cell • NCI-H1792 cell • HeLa cell • HEK293

Machine-accessible metadata file describing the reported data (ISA-Tab format)

## Background & Summary

Developing novel therapies for targeting RAS-driven cancers has proven difficult, due to the complex redundancies and feedback mechanisms in the RAS pathway and challenges targeting mutated RAS proteins directly^1^. To this end, the National Cancer Institute (NCI)’s RAS Initiative (https://www.cancer.gov/research/key-initiatives/ras) has focused attention and resources to understanding RAS-related biology and to discovering therapies for RAS-driven cancers. One of the major goals of the RAS Initiative is to develop reagents and assays to enable the next generation of RAS drug discovery^2^. This aligns with one of the missions of the NCI’s Office of Cancer Clinical Proteomics Research (OCCPR), that is, to provide publicly accessible, well-characterized reagents and resources for the cancer research community. As a result, the NCI-OCCPR and the NCI-RAS Initiative jointly launched a proteomic assay development and characterization project targeting RAS-associated pathways. The goal is to develop, validate, and distribute monoclonal antibodies enabling enrichment and/or detection of proteins and post-translational modifications involved in RAS signal transduction.

While standardized procedures for antibody validation have yet to be developed, a consensus set of guidelines has been presented^3^, as well as ‘consensus principles’^4^. These guidelines suggest that fit-for-purpose validation of antibodies depends on the intended application and should generally ascertain the antibody specificity and sensitivity for the target of interest. Demonstration of the usefulness of the antibody in the intended application should be provided using standards and/or real samples wherever possible. Finally, transparency in the protocols and techniques used to validate antibodies is useful for researchers seeking to apply the reagents in their own research.

Herein, we report the validation of a suite of 104 novel monoclonal antibodies (mAbs) targeting 27 phosphopeptides and 69 unmodified peptides, to 20 proteins involved in the RAS network, with the validation being conducted under the ‘consensus principles’^4^. Validation datasets are presented for applications in Western blotting, protein immunoprecipitation, protein arrays, immunohistochemistry (IHC), and peptide immunoaffinity enrichment, as well as the success rates for generation of antibodies across these applications. The corresponding datasets and antibody reagents are available on the CPTAC Antibody Portal (https://antibodies.cancer.gov/), the Panorama Public repository^5,6^, the PRIDE proteomics database^7,8^, and ProteomeXchange^9,10^. The characterization dataset and antibody reagents are of value to the cancer research community in providing new resources to advance the understanding of the RAS biological network and discovery of therapies against RAS-driven cancers.

**Fig. 1:**
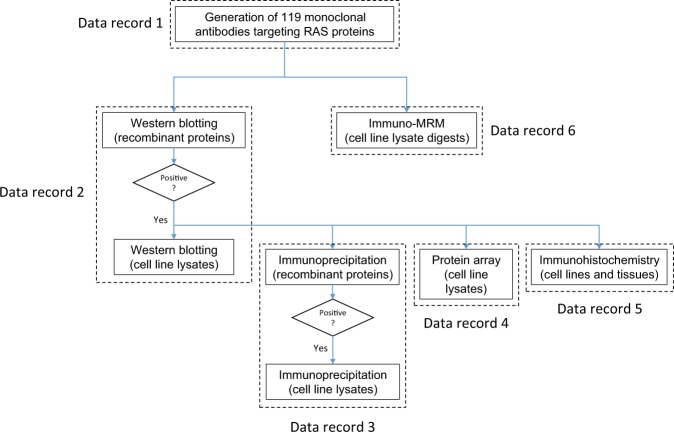
Overview of the datasets describing validation of a novel suite of mAbs.

A summary of the antibodies and associated validation datasets is shown in Fig. 1. The mAbs were generated in mice and rabbits using proteotypic^11^ peptide immunogens from 21 RAS network proteins. Hybridomas were screened by peptide ELISA as well as immuno-MRM to identify clones producing monoclonal antibodies for the target immunogen peptide. A total of 119 monoclonal antibodies were produced and affinity-purified, of which we successfully validated 104 antibodies in at least one application. Antibodies were first tested by Western blotting using recombinant proteins, and positive antibodies in Western blots were evaluated in four arms, (i) Western blotting of cell line lysates, (ii) protein immunoprecipitation of recombinant proteins and subsequently in cell line lysates, (iii) protein array detection in cell line lysates, and (iv) immunohistochemistry in cell lines and tissues. In a separate characterization arm, the best mAb to a given peptide and/or modification site was evaluated for detection of endogenous signal from cell line lysates by peptide immunoaffinity enrichment and targeted mass spectrometry (immuno-MRM). A summary of the number of antibodies tested and found to support each of the applications is presented in Table 1. By Western blotting, 63 (53%) of the 119 mAbs were positive against recombinant proteins, and 41 of these 63 mAbs (34% of the 119 mAbs) were positive in cell lines. In IP-MS experiments, 56 mAbs captured recombinant protein and 15 of the 56 mAbs captured endogenous protein in cell line lysates (47% and 13% of 119 mAbs, respectively). The protein array analyses encompassed testing against the NCI-60 cell line collection (https://dtp.cancer.gov/discovery_development/nci-60/default.htm), and 17 (14%) of the 119 mAbs were positive in these cell lines. Finally, immunohistochemistry analyses using the NCI-60 cell lines and four cancer tissues showed that 27 mAbs (23%) and 25 mAbs (21%) were positive, respectively. For immuno-MRM, 19 (70%) of the 27 phosphopeptides and 57 (83%) of the 69 unmodified peptides targeted were detected by endogenous immuno-MRM in the cell lines under the conditions tested.

**Table 1:** Summary of data records associated with validation of the novel suite of antibodies.

Data record	Data record location	Application	# Antibodies tested	# Antibodies positive	Test matrix
2	‘Western blot data’^12^, and the ‘Western Blot Results Summary Table’ in ‘Results Summary Tables and Data Tables’^12^	Western blotting	119	63	recombinant protein
2	‘Western blot data’^12^, and the ‘Western Blot Results Summary Table’ in ‘Results Summary Tables and Data Tables’^12^	Western blotting	63	41	cell lines
3	PRIDE^8^; the ‘IP-MS Results Summary Table’ and the ‘IP-MS Peptide and Protein Intensity Table’ in ‘Results Summary Tables and Data Tables’^12^	Immunoprecipitation	61	56	recombinant protein
3	PRIDE^8^; the ‘IP-MS Results Summary Table’ and the ‘IP-MS Peptide and Protein Intensity Table’ in ‘Results Summary Tables and Data Tables’^12^	Immunoprecipitation	57	15	cell lines
4	‘Protein array data’^12^; the ‘Protein Array Results Summary Table,’ the ‘Protein Array Raw Data Table,’ and the ‘Protein Array Normalized Data Table’ in ‘Results Summary Tables and Data Tables’^12^	Protein array	62	17	cell lines
5	‘Tissue immunohistochemistry data’^12^, and the ‘Immunohistochemistry Results Summary Table’ in ‘Results Summary Tables and Data Tables’^12^	Immunohistochemistry	54	27	cell lines and tissues
6	Panorama Public Repository^6^, ProteomeXchange^10^, and the ‘Immuno-MRM Results Table’ in ‘Results Summary Tables and Data Tables’^12^	Immuno-MRM	104	89	cell lines

## Methods

### Monoclonal antibody generation

Target proteins were identified by the NCI’s RAS Initiative and include high priority proteins and modifications involved in the RAS signaling network. All antibodies were generated to peptide sequences with lengths between 7 and 31 amino acids (see the ‘Antibody Summary Table’ in ‘Results Summary Tables and Data Tables’^12^). Synthetic peptides used as antigens contained an additional cysteine residue on either the N- or C-terminus to facilitate chemical coupling. All internal cysteines were carbamidomethylated.

Custom mouse mAbs to unmodified peptide sequences were generated by Precision Antibody (Columbia, MD). Swiss Webster derived mice (SJL strain; females 5–7 weeks old) were immunized with the target peptide antigen, and titers were determined by ELISA against immobilized peptide antigen. Once a sufficient titer was detected, splenocytes were harvested and fused with a murine myeloma partner using a high efficiency electrofusion procedure. Direct cloning was performed in a semi-solid, HAT selection medium prior to screening, utilizing an automated clone picking system. Supernatant from the resulting clonal hybridomas were screened by ELISA against the target antigen and a counter-screening antigen. Positive clones were expanded and cryopreserved. Antibody concentrations in the positive supernatants were determined by ELISA as previously described^13^, except using AffiniPure goat-anti-mouse IgG (subclasses 1 + 2a + 2b + 3), Fcγ fragment specific (115-005-164, Jackson ImmunoResearch, West Grove, PA) as the capture antibody, and horseradish peroxidase AffiniPure goat-anti-mouse IgG, F(ab’)_2_ fragment specific (115-035-072, Jackson ImmunoResearch) as the detection antibody. The positive clones by ELISA were then screened using immuno-MRM.

Immuno-MRM screening was performed using sheep-anti-mouse IgG magnetic beads (112-02D, Invitrogen, Carlsbad, CA) to couple up to 1.0 μg of mouse mAb (from supernatants). Each mAb supernatant was tested separately. A negative control contained 100 μL of 1X PBS-0.01% CHAPS and 100 μL of hybridoma growth medium (no mAb). The antibody-coupled beads were used to capture between 200 and 2000 fmol of light synthetic peptide (New England Peptide, Gardner, MA) in a background matrix of 10 μg digested MCF10A lysate. After washing in PBS/0.01% CHAPS, the peptides were eluted in 25 μL of 3% acetonitrile-5% acetic acid-50 mM citrate containing heavy synthetic stable isotope-labeled peptides (at the same amount as the light peptides). All magnetic bead handling was performed using a KingFisher 96 magnetic bead handling platform (5400500, Thermo Scientific) equipped with a PCR magnet head. The elution plate was covered with chemically resistant sealing foil (T-3025-8B, BioExpress/VWR, Radnor, PA) and frozen at −80 °C until analysis by LC-MRM-MS. 10 μL of an eluate sample was injected per LC-MRM-MS experiment. The data were analyzed using Skyline^14^, and the peak area ratio (light:heavy) was used to select the best one to four mAbs per peptide for production and purification.

Custom rabbit hybridoma mAbs to phosphopeptide antigens were generated by Abcam (Cambridge, MA). Each phosphopeptide antigen was conjugated to KLH, ovalbumin, and blue carrier protein using SMCC, and mixed with Freund’s adjuvant. For each phosphopeptide antigen, two New Zealand white rabbits were immunized with KLH-conjugated peptide, followed by two injections with ovalbumin-conjugated peptide. The titers were tested using a peptide-MBS-BSA ELISA (MBS, m-maleimidobenzoyl-N-hydroxysuccinimide ester), and the best rabbit of a pair was immunized for a final boost using blue carrier protein-conjugated peptide, followed by splenectomy and hybridoma generation. The hybridoma primary clone supernatants were tested by peptide-MBS-BSA ELISA, and the mAb concentrations of positive supernatants were measured by ELISA as previously described^15^. The positive supernatants were then screened by immuno-MRM analogously to the mouse hybridomas, with the following main differences: (i) the magnetic beads used were sheep-anti-rabbit IgG beads (112-04D, Invitrogen), and (ii) up to 0.5 μg of rabbit mAb was used. Up to two best phospho-specific and two best total mAbs (the latter recognizing both the phosphorylated and unmodified peptides) per peptide target were advanced to subcloning, and peptide-MBS-BSA ELISA-positive subclones were screened by immuno-MRM. Up to one best phospho-specific and two best total subclones per immunogen peptide were moved to production and purification. The selected hybridoma mAbs were cloned by LakePharma (San Carlos, CA) and Excel BioPharm (Burlingame, CA). A list of the mouse and rabbit antibodies that were generated and subsequently validated in at least one application can be found in Data Record 1 (see the ‘Antibody Summary Table’ in ‘Results Summary Tables and Data Tables’^12^).

### Cell lines and lysate generation

Commercially available cell lines were obtained from the American Type Culture Collection (Manassas, VA). These included: the human mammary epithelial cell line MCF10A (CRL-10317); the human lung cancer cell lines H2122 (CRL-5985), H2444 (CRL-5945), H1792 (CRL-5895), and A549 (CCL-185); the human pancreatic cancer cell lines BxPC-3 (CRL-1687) and AsPC-1 (CRL-1682); the human cervical cancer cell line HeLa (CCL-2); and the human embryonic kidney cell line HEK293 (CRL-1573). The MCF10A isogenic cell line stably transfected with a vector containing the activated G12V mutation in the KRAS gene (MCF10A-KRAS) and a control vector for wild type^16^ (MCF10A-empty vector) were obtained from Dr. Sourav Bandyopadhyay’s laboratory (UCSF, San Francisco, CA). All of the cell line identities were confirmed by DNA fingerprinting using STR (Short Tandem Repeats) CODIS (Combined DNA Index System) typing (below). The following growth media were used for the cell lines: MCF10A – DMEM/F12 1:1 (11320, Gibco) supplemented with sterile filtered 5% horse serum (16050, Gibco), 10 μg/mL of insulin (I6634, Sigma, St. Louis, MO), 20 ng/mL of EGF (AF-100-15, PeproTech, Rocky Hill, NJ), 0.5 μg/mL of hydrocortisone (H0888, Sigma), 100 ng/mL of Cholera toxin (C8052, Sigma), and 100 units/mL of penicillin-streptomycin (15140122, Gibco); H2122, H2444, H1792, A549, BxPC-3, and AsPC-1 – RPMI-1640 (11875, Gibco) supplemented with 10% heat-inactivated fetal bovine serum (FBS) (SH30071.03, Hyclone, or 16140071, Gibco), 1 mM sodium pyruvate (11360, Gibco), and 100 units/mL of penicillin-streptomycin (15140, Gibco); HeLa and HEK293 – Eagle’s Minimum Essential Medium (10370, Gibco) supplemented with 10% heat-inactivated FBS (SH30071.03, Hyclone), 1 mM sodium pyruvate (11360, Gibco), 2 mM L-glutamine (25030, Gibco), and 100 units/mL of penicillin-streptomycin (15140, Gibco). All cell cultures were incubated at 37 °C, 5% CO_2_. As the HeLa and HEK293 cells approached 80% confluency, the growth medium was removed and cells were rinsed twice with D-PBS (14190, Gibco) then replaced with starvation medium (growth medium without FBS) overnight. The next day, HeLa cells were stimulated with 50 ng/mL of EGF (AF-100-15, PeproTech) for 10 min in a 37 °C, 5% CO_2_ incubator and HEK293 cells were treated with 100 nM of insulin (I6634, Sigma) for 10 min in a 37 °C, 5% CO_2_ incubator before the cells were harvested. The remaining cells were cultured to 80–90% confluency in regular growth medium before the cells were harvested.

At the end of the incubation time, EGF-stimulated HeLa cells and insulin-stimulated HEK293 cells were taken out of the 37 °C, 5% CO_2_ incubator, medium was removed, and adherent cells were rinsed with D-PBS. Freshly-prepared ice-cold urea lysis buffer (containing 6 M urea (U0631, Sigma), 25 mM Tris (pH 8.0) (T2194, Sigma), 1 mM EDTA (E7889, Sigma), 1 mM EGTA (E0396, Sigma), 1% phosphatase inhibitor cocktail 2 (P5726, Sigma), 1% phosphatase inhibitor cocktail 3 (P0044, Sigma), and 1% protease inhibitor cocktail (P3840, Sigma)) was added directly to the attached cells and a cell lifter (3008, Corning, Tewksbury, MA) was used to scrape the cells off the plate. This cell lysate suspension was then transferred to a pre-cooled 50 mL tube and sonicated twice for 12 seconds using a Sonic Dismembrator (Fisher, Model 100) at setting level 1. Lysates were transferred to microcentrifuge tubes, vortexed, and then cleared by centrifugation at 20,000 × g for 10 min at 4 °C. Supernatants were transferred to cryo-vials and stored in liquid nitrogen until use. The remaining cells (H2122, H2444, H1792, A549, BxPC-3, AsPC-1, MCF10A, MCF10A-KRAS, and MCF10A-empty vector) were harvested by removing the medium, rinsing adherent cells with D-PBS or warm 0.05% Trypsin-EDTA solution (25300062, Gibco), and detaching the cells from the culture vessel by adding warm 0.25% or 0.05% Trypsin-EDTA solution (25200 or 25300062, respectively, Gibco) and incubating for 3–10 min at room temperature until the cells had lifted (cells that were difficult to detach were incubated at 37 °C, 5% CO_2_). Trypsin was inactivated by adding base growth medium with 5% horse serum or 10% FBS and the cells were transferred to pre-cooled 50 mL tubes, spun at 400 × g for 8 min at 4 °C to remove the medium, and washed twice with ice-cold D-PBS buffer. Freshly-prepared ice-cold urea lysis buffer (above) was added to the cell pellets and sonicated, cleared, and stored as above. The cell lysate protein concentration was measured using the Micro BCA Protein Assay Kit (23235, Thermo, Waltham, MA).

### DNA fingerprinting and short tandem repeat (STR) profiles

Short tandem repeat (STR) profiles were generated for each of the cell lines. Allele profiles are as follows:

MCF10A-KRAS and MCF10A-MT: Amelogenin: X; CSF1PO: 10, 12; D13S317: 8, 9; D16S539: 10, 12; D18S51: 18, 19; D19S433: 13, 15; D21S11: 28, 30; D2S1338: 21, 26; D3S1358: 14, 18; D5S818: 10, 13; D7S820: 10, 11; D8S1179: 14, 16; FGA: 22, 24; TH01: 8, 9.3; TPOX: 9, 11; vWA: 15, 17

H2122: Amelogenin: X; CSF1PO: 10; D13S317: 10; D16S539: 9, 12; D18S51: 19; D19S433: 14; D21S11: 33.2; D2S1338: 17; D3S1358: 15, 16; D5S818: 11, 12; D7S820: 8, 10; D8S1179: 13; FGA: 19, 26; TH01: 7, 9.3; TPOX: 9; vWA: 17

H2444: Amelogenin: X; CSF1PO: 12; D13S317: 11, 12; D16S539: 12; D18S51: 13; D19S433: 16, 17; D21S11: 28, 29; D2S1338: 20; D3S1358: 14; D5S818: 10; D7S820: 8, 10; D8S1179: 12, 13; FGA: 25; TH01: 9.3; TPOX: 11; vWA: 17

H1792: Amelogenin: X; CSF1PO: 10; D13S317: 12; D16S539: 10, 11; D18S51: 15, 17; D19S433: 14; D21S11: 31; D2S1338: 19; D3S1358: 15; D5S818: 12; D7S820: 8, 11; D8S1179: 12, 15; FGA: 22, 24; TH01: 7; TPOX: 11; vWA: 14, 16

A549: Amelogenin: X, Y; CSF1PO: 10, 12; D13S317: 11; D16S539: 11, 12; D18S51: 14, 17; D19S433: 13; D21S11: 29; D2S1338: 24; D3S1358: 16, 20; D5S818: 11; D7S820: 8, 11; D8S1179: 13, 14; FGA: 23; TH01: 8, 9.3; TPOX: 8, 11; vWA: 14

BxPC-3: Amelogenin: X; CSF1PO: 13; D13S317: 11; D16S539: 9, 11; D18S51: 12; D19S433: 13, 16.2; D21S11: 29; D2S1338: 17, 19; D3S1358: 14, 16; D5S818: 11; D7S820: 10, 13; D8S1179: 13; FGA: 20, 21; TH01: 9; TPOX: 8; vWA: 14, 18

AsPC-1: Amelogenin: X; CSF1PO: 10, 13; D13S317: 9, 12; D16S539: 11; D18S51: 18; D19S433: 14; D21S11: 28, 30; D2S1338: 22, 23; D3S1358: 16; D5S818: 12; D7S820: 12, 13; D8S1179: 13, 15; FGA: 24; TH01: 7, 9.3; TPOX: 8, 10; vWA: 17

HeLa: Amelogenin: X; CSF1PO: 9, 10; D13S317: 12, 14; D16S539: 9, 10; D18S51: 16; D19S433: 13, 14; D21S11: 27, 28; D2S1338: 17; D3S1358: 15, 18; D5S818: 11, 12; D7S820: 8, 12; D8S1179: 12, 13; FGA: 18, 21; TH01: 7; TPOX: 8, 12; vWA: 16, 18

HEK293: Amelogenin: X; CSF1PO: 11, 12; D13S317: 12; D16S539: 9, 13; D18S51: 17; D19S433: 15, 18; D21S11: 28, 30.2; D2S1338: 19; D3S1358: 15; D5S818: 8, 9; D7S820: 11, 12; D8S1179: 12, 14; FGA: 23; TH01: 7, 9.3; TPOX: 11; vWA: 16, 19

### Validation of antibodies in Western blotting

Western blot analyses were performed using recombinant proteins, and mAbs that were positive in these experiments were subsequently tested using cell line lysates. Recombinant proteins were obtained from Origene (Rockville, MD) and Novus Biologicals (Littleton, CO); see ‘Western blot data’^12^ for catalog numbers. MCF10A-KRAS and BxPC-3 cell lines were cultured and lysed as above, with the following modifications. Cell pellets (~5 × 10^7^ cells) were suspended in 1 mL of M-PER (Mammalian Protein Expression Reagent, 78501, Pierce) and then mechanically disrupted in a small Dounce homogenizer (using 5 up & down strokes). Lysates were then centrifuged at 1700 × g for 10 min (4000 rpm in an Eppendorf 5810R centrifuge using a F45-30-11 rotor). Pellets were discarded, and supernatants were analyzed for protein concentration using a standard Bradford assay. Western immunoassays were performed using the Wes system (ProteinSimple, San Jose, CA) according to the manufacturer’s protocol and as described previously^17^, with the following modifications. Fluorescent Master Mix was prepared using 1 part 5x Fluorescent Mix and 5 parts protein preparation (recombinant protein at 1 μg/mL, lysates at 200 μg/mL), and 2 ng of recombinant protein or 400 ng of lysate were injected per experiment. Primary antibodies were diluted in 1X PBS to a concentration of 1 μg/mL, then diluted 1:5 in 1x SignaLOCK Blocking Solution (50-58-00, SeraCare, Milford, MA). The Antibody Diluent Time was changed from 5 min to 30 min, and the Detection Profile was changed from 7 exposures to 8 exposures (1, 5, 15, 30, 60, 120, 240, and 480 seconds). Data from antibody validation by Western blotting are found in Data Record 2 (see the ‘Western Blot Results Summary Table’ in ‘Results Summary Tables and Data Tables’^12^ and ‘Western blot data’^12^).

### Validation of antibodies in protein immunoprecipitation (IP)

Protein IP-MS analyses were performed by Poochon Scientific (Frederick, MD) on 61 mAbs that were positive by Western blotting against recombinant proteins. The antibodies were first tested by IP-MS against recombinant proteins by grouping the antibodies into nine multiplexed groups with six or seven mAbs per group (see the ‘IP-MS Results Summary Table’ in ‘Results Summary Tables and Data Tables’^12^). Fifty-six mAbs with positive immunoprecipitation using recombinant proteins were subsequently tested by IP-MS using MCF10A-KRAS and MCF10A-empty vector cell lysates.

The MCF10A-KRAS and MCF10A-empty vector cells were grown as described above and lysed as follows. Total protein was extracted from each cell type by adding 1.0 mL of ice-cold RIPA lysis buffer (89900, Thermo Fisher Scientific) containing 10 μL of freshly-added protease inhibitor cocktail (P8340-5 mL, Sigma) to a cell pellet (12 × 10^7^ cells) and incubating on ice for 30 min. The cells were further disrupted and homogenized by sonication (10 cycles, 10 seconds on and 10 seconds off and 15% output per cycle) using Fisher Scientific Sonic Dismembrator model 500, while taking care not to raise the temperature of the lysate, followed by 30 min of incubation on ice. The lysates were centrifuged at 15,300 × g for 15 min at 4 °C, and the supernatant was transferred to a new microcentrifuge tube. The protein concentration was determined using BCA Reducing Reagent compatible assay kit (Thermo Fisher Scientific). The MCF10A-KRAS and MCF10A-empty vector lysates’ protein concentrations were 4.95 mg/mL and 3.83 mg/mL, respectively.

Each multiplexed mAb IP group (5 μg per antibody) was incubated with a master mix of the 19 recombinant proteins (0.5 μg per protein), or with 1 mL of each cell lysate in PBS-0.1% Tween-20 for 2 hours at 4 °C. Following incubation, 50 μL of Protein A/G agarose beads (1861760, Pierce) was added to each IP group tube, and the mixtures were incubated overnight at 4 °C. The beads were pelleted by centrifugation at 110 × g for 3 min, and the supernatants were discarded. The bead pellets from the IPs using recombinant proteins were washed 3 times with 1X PBS containing 0.2% Tween-20, with repeating the centrifugation step each time. IPs from cell lysates were washed with 1X RIPA buffer. After the final wash, the supernatants were aspirated and discarded, and the pellets were re-suspended in 40 μL of 2X electrophoresis sample buffer (NuPAGE LDS Sample Buffer, NP0007, Invitrogen/Life Technologies, 100 mM Tris pH 6.8, 40 mM DTT, 2% SDS, 20% glycerol, 0.2% bromophenol blue). Negative controls consisted of 50 μL of the Protein A/G beads and the 19-protein master mix (0.5 μg per protein) or cell lysates without adding antibodies.

Each sample in LDS sample buffer was heated at 95 °C for 10 min and fractionated using SDS-PAGE (NuPAGE™ 4–12% Bis-Tris protein gel, NP0322BOX, Life Technologies) with a MES SDS running buffer. Each IP sample was split over 2 gel lanes, yielding two sample processing duplicates from this point forward. The gels were stained with SimplyBlue™ SafeStain (LC6065, Life Technologies) for 1 hour with gentle shaking and then washed with deionized water. All protein bands were cut out of the gel, diced into 1–2 mm^2^ pieces, and placed into a 1.5 mL centrifuge tube. The gel pieces were de-stained by washing two times in 100 μL of 25 mM NH_4_HCO_3_/50% acetonitrile for 10 min at room temperature and discarding the supernatant. The gel pieces were completely dried using a SpeedVac, and then reduced using 10 mM DTT in 25 mM NH_4_HCO_3_ (enough to cover the gel pieces) at 56 °C for 1 hour. After cooling to room temperature, the supernatants were removed and 50 μL of 55 mM iodoacetamide was added to the gel pieces and incubated in the dark for 45 min at room temperature. The supernatants were discarded, and the gel pieces were washed by adding 25 mM NH_4_HCO_3_ (enough to cover the gel pieces) and incubating for 10 min at room temperature. The supernatants were removed and the gels were dehydrated twice by adding 25 mM NH_4_HCO_3_/50% acetonitrile (enough to cover) and then incubating for 10 min at room temperature. The gel pieces were then dried to completeness using a SpeedVac. The samples were then enzymatically digested by adding 12.5 ng/μL trypsin (Pierce™ Trypsin Protease, MS Grade, 90057, Thermo Fisher Scientific) in 25 mM NH_4_HCO_3_ to each tube (enough to cover the gel pieces) and rehydrating the gel pieces for 30 min at 4 °C. Excess trypsin solution was removed to reduce the amount of auto-digested trypsin in the final samples, and the gel pieces were washed briefly with 50 μL of 25 mM NH_4_HCO_3_. The wash solution was discarded, the gel pieces covered with 25 mM NH_4_HCO_3_, and the samples incubated overnight at 37 °C. The protein digest supernatants were then transferred to clean 1.5 mL centrifuge tubes, and 50% acetonitrile/0.5% formic acid was added to the left-over gel pieces (enough to cover them). The gel pieces were incubated at room temperature for 30 min, and then the supernatants were pooled with their respective protein digest supernatants. The digests were dried to 5–10 μL using a SpeedVac, and then 15 μL of 0.1% formic acid was added to each digest. The digests were desalted using C18 Zip-Tips (ZTC18S960, Millipore) before LC-MS/MS analysis. For LC-MS/MS, the final samples were in 20 μL of 0.1% formic acid, and 16 μL was injected per LC-MS/MS analysis. A positive control consisted of a mix of the 19 protein targets (25 ng/target) that was digested the same way.

The LC-MS/MS analyses were carried out using a Thermo Scientific Q-Exactive hybrid Quadrupole-Orbitrap Mass Spectrometer and a Thermo Dionex UltiMate 3000 RSLCnano System. Each IP digest (16 μL) was loaded onto a peptide trap cartridge column (160454, Thermo Fisher Scientific) at a flow rate of 5 μL/min. The trapped peptides were eluted onto a reversed-phase 20 cm C18 PicoFrit column (ProteoPep 2, New Objective) using a linear gradient of acetonitrile (3–36%) in 0.1% formic acid over 60 min at a flow rate of 0.3 μL/min. The Q-Exactive was equipped with a Nanospray Flex Ion Source ES071 (Thermo Fisher Scientific), and a spray voltage of 1.8 kV and a capillary temperature of 250 °C were applied. The Q-Exactive instrument was operated in the data dependent mode to automatically switch between full scan MS and MS/MS acquisition. Survey full scan MS spectra (m/z 300–1800) were acquired in the Orbitrap with the resolution set to 70,000 (m/z 200) after accumulation of ions to a 1 × 10^6^ target value based on predictive automatic gain control (AGC). Dynamic exclusion was set to 20 seconds. The 15 most intense multiply charged ions (z ≥ 2) were sequentially isolated and fragmented in the octopole collision cell by higher-energy collisional dissociation (HCD) using a normalized HCD collision energy of 28% with an AGC target of 1 × 10^5^ and a maximum injection time of 100 milliseconds at a 17,500 resolution.

Raw MS/MS spectra from the analysis were searched against reviewed Human Universal Protein Resource (UniProt) sequence database release 2016_01 using MaxQuant/Andromeda^18^. The search was performed with tryptic enzyme constraint set for up to two missed cleavages, oxidized methionine, phosphorylated serine, threonine, and tyrosine set as variable modifications, and carbamidomethylated cysteine set as a static modification. Peptide MH+ mass tolerances were set at 20 ppm. The overall peptide FDR was set at ≤1%. Data from immunoprecipitation characterization by LC-MS can be found in Data Record 3^8^ (see also the ‘IP-MS Results Summary Table’ and the ‘IP-MS Peptide and Protein Intensity Table’ in ‘Results Summary Tables and Data Tables’^12^).

### Validation of antibodies by protein array analysis

Protein array analyses were performed using NCI-60 cell lines obtained from the Cancer Research Technology Program at NCI-Frederick and the MCF10A-KRAS cell line. 62 purified monoclonal antibodies that had been positive by Western blotting using recombinant proteins were tested in the protein array analyses (one mAb had not been cloned when these protein array experiments were performed, hence it was not tested here). The NCI-60 and MCF10A-KRAS cell lines were collected at the log phase growth and protein prepared by resuspending cell pellets in T-PER (78510, Pierce Biotechnology, Rockford, IL) per the manufacturer’s recommendations; total protein concentration was measured by BCA Protein Assay kit (23225, Pierce). Quantification of protein expression values was performed by well-based reverse phase protein array (RPPA) as previously reported^19,20^. Briefly, five microliters (2000 ng-50 ng/well) of MCF10A-KRAS or NCI-60 cell line antigens in PBST (1X PBS, 0.1% Tween-20) were applied onto 96-well Multi-Spot™ plates (MA2400 96 HB Plate, Meso Scale Discovery, Gaithersburg, MD). The plates were allowed to dry at room temperature for 2 hours. Prior to primary antibody incubation, the antigen-coated plates were blocked with 5% non-fat dry milk in PBST for 1 hour at room temperature. Target-specific antibodies were diluted (160–240 ng/mL) with 5% BSA in PBST. For each cell line, 25 μL of antibody were added and incubated overnight at 4 °C. The plates were washed with PBST and followed by a 90 min incubation with goat anti-mouse or anti-rabbit SULFO-TAG™ antibodies (Meso Scale Discovery) at a dilution of 1:2000 (0.5 µg/mL) containing 5% non-fat dry milk in PBST. Plates were washed, and MSD-T read buffer was added to the plate to detect binding signals using Sector Imager 2400 reader (Meso Scale Discovery). PBST-coated wells were included on each plate as a control of non-specific binding. For each mAb, the electrochemical luminescence value of each cell line was normalized by the average value of the 60 cell lines, and the resulting normalized values were in turn normalized to a mean equal to 1.0 and a standard deviation of 0.5. The resulting values are plotted in bar graphs (see ‘Protein array data’^12^). Based on the normalization that had set the standard deviation to 0.5, detection of a protein in a cell line was deemed positive, positive (weak), or negative if the normalized value was above 1.5, between 0.5 and 1.5, or below 0.5, respectively, indicated by green, grey, and red bars in the bar graphs. Final antigen-antibody concentration was established through preliminary testing (data not shown). Only antibodies that showed a linear correlation of signal expression and protein concentration in the MCF10A-KRAS control cell line were screened against the entire NCI-60 panel. Data from protein array characterization can be found in Data Record 4 (see the ‘Protein Array Results Summary Table,’ the ‘Protein Array Raw Data Table,’ and the ‘Protein Array Normalized Data Table’ in ‘Results Summary Tables and Data Tables’^12^, and ‘Protein array data’^12^).

### Validation of antibodies in cell and tissue immunohistochemistry

54 purified cloned monoclonal antibodies that tested positive by Western blotting using recombinant proteins were tested in the IHC analyses (eight rabbit mAbs that had been negative in the protein array analyses were not tested by IHC). NCI-60 cell microarray (CMA) and breast, ovary, colon, and lung tissue microarray (TMA, MTA-6A^20^) blocks were constructed using a tissue arrayer (Pathology Devices, Westminster, MD). Briefly, slides were reviewed by a pathologist, and areas containing a desired category were annotated on the hematoxylin and eosin (H&E) slides. Cylindrical tissue cores of 1.0 mm diameter were then taken from the corresponding regions of the paraffin blocks and transplanted into a recipient paraffin block.

For IHC staining, all paraffin-embedded CMA/TMA sections were cut at 5-micron thickness, deparaffinized through xylene and rehydrated with graded ethanols. Antigen retrieval was performed with a steamer (Black & Decker, Towson, MD) with heat induced epitope retrieval pH 10.0 citrate buffer (S3307, Dako, Carpinteria, CA) for 20 min at 80 °C. Endogenous peroxidase activity was blocked with 3% H_2_O_2_ for 10 min followed by primary antibody incubation at optimized dilution (above) for 2 hours at room temperature. Subsequently, sections were labeled with biotinylated link secondary enzyme conjugated streptavidin (LSAB) (K0675, Dako) for 30 min, visualized with 3,3-diaminobenzadine (K3468, Dako) for 10 min, hematoxylin (72711, Thermo Fisher Scientific) counterstained, washed, dehydrated with anhydrous ethanols, cleared in xylene, and coverslipped. (The slides were stained using an automated system (Dako Autostainer model LV-1), with the machine dispensing 300 μL of solution per slide, then counterstained using a Leica Autostainer model XL (Leica Biosystems, Buffalo Grove, IL).) Negative control samples were processed the same way, yet without primary antibody.

IHC evaluation was based on the subcellular localization (nuclear, cytoplasmic, membranous, and/or combination) in the cell type of interest and the overall intensity of staining. Staining was characterized as none, weak, moderate, and strong for each core. Optimum antibody titration was defined as maximum dynamic range of signal (negative to positive) across the diversity of cell lines at the minimum dilution to achieve this staining pattern. Based on the NCI-60 CMA titration scale, similar conditions were used to stain the tissue array MTA-6A. Data from immunohistochemistry characterization can be found in Data Record 5 (see the ‘Immunohistochemistry Results Summary Table’ in ‘Results Summary Tables and Data Tables’^12^; tissues that had positive staining are illustrated in ‘Tissue immunohistochemistry data’^12^).

### Characterization of endogenous peptide detection by immuno-MRM

The detection of endogenous peptides by immuno-MRM was tested in digested protein lysates from a combination of the following cell lines (above): (1) a 1:1:1:1 pool (by protein mass) of H2122, H2444, BxPC-3, and AsPC-1 (termed “4 RAS cell line mix” in the ‘Immuno-MRM Results Table’ in ‘Results Summary Tables and Data Tables’^12^); (2) MCF10A-KRAS; (3) A549; (4) H1792; (5) EGF-stimulated HeLa; and (6) insulin-stimulated HEK293. For testing mouse mAbs to unmodified peptide targets, an input of 75 or 100 μg of digested lysate was used (indicated in the ‘Immuno-MRM Results Table’ in ‘Results Summary Tables and Data Tables’^12^); for testing rabbit mAbs and for testing mouse mAbs that can capture phosphopeptides (see Data Record 6 below), 500 μg of digested lysate was used. Enzymatic digestion was performed as follows. Lysates were reduced by adding TCEP (77720, Thermo) to a final concentration of 6% (v/v), with incubation at 37 °C for 30 min with shaking. Reduced lysates were alkylated by iodoacetamide (IAM, A3221, Sigma, or 90034, Pierce) at a final concentration of 40 mM with incubation in the dark at room temperature for 30 min. The mixture was diluted 1:10 with 100 mM Tris, pH 8 (T2694, Sigma) and sequencing-grade trypsin (V511X, Promega, Madison, WI) was added at a ratio of 1:50 (enzyme:protein mass), and incubated at 37 °C with shaking. After two hours, a second trypsin aliquot was added at a 1:100 ratio (enzyme:protein mass) and incubated overnight at 37 °C with shaking. The trypsin activity was then quenched with 20% formic acid (1.11670.1000, EMD Millipore, Burlington, MA) for a final concentration of 1% by volume. Digested cell lysates were desalted on Oasis HLB 96-well plates (60 mg, 186000679 or 30 mg, WAT058951, Waters, Milford, MA) using a Positive Pressure-96 Processor Manifold (186006961, Waters) and peptides were eluted from the plates with 3 × 0.4 mL of 0.1% formic acid in 50% acetonitrile. The digests were lyophilized and resuspended in 1X PBS (BP399-20, Fisher) + 0.01% CHAPS (28300, Thermo Scientific) to a concentration of 2.5 mg/mL, and 1 M Tris, pH 8.0 (T2694, Sigma) was added to adjust the pH to 7–8.

For immunoaffinity enrichment, the mAbs were coupled to 1 μm Protein G magnetic beads (custom-made, Dynabeads® MyOne Protein G, supplied as 30 mg/mL in PBS with 0.02% sodium azide, Life Technologies) at an antibody-to-bead ratio of 1.0 μg antibody to 2.0 μL beads. Immunoaffinity enrichment using hybridoma supernatants or purified mAbs were performed similarly as described for the screening experiments (above). Negative control samples for all endogenous characterization experiments were prepared using the same digest amounts and bead volumes with no mAbs.

Detection of the target peptides in the elution buffer of the enriched samples was performed by positive ion multiple reaction monitoring mass spectrometry on a QTRAP mass spectrometer (Sciex) interfaced to an Eksigent nano-HPLC system. Mobile phases A and B consisted of 0.1% formic acid (1.11670.1000, EMD Millipore) in water (W6-4, Fisher Scientific), and 0.1% formic acid in 90% acetonitrile (A955–4, Fisher Scientific) in water, respectively. Peptides were loaded onto a trap column (Acclaim PepMap 100 C18, 5 μm, 100 Å, 160454, Dionex, Sunnyvale, CA; or ReproSil-PUR C18AQ, 3 μm, 804-00016, AB Sciex) and eluted from the nano-analytical column (ReproSil-PUR C18-AQ, 3 μm, Dr. Maisch GmbH, Ammerbuch, Germany; or ChromXP C18-CL, 3 μm, 804-00001, AB Sciex) using a linear gradient of 0-60% B at 300 nL/min. The mass spectrometer was operated in positive ion mode. The resolutions for Q1 and Q3 were set to Unit, the settling time was set to 0 msec, and the pause between mass ranges was set to 3.000 msec. Methods consisted of unscheduled MRM acquisition if they contained less than ~300 transitions. Transitions were scheduled if the number exceeded ~300 total transitions in a method. Data from antibody characterization of endogenous detection by immuno-MRM can be found in Data Record 6^6,10^ (see also the ‘Immuno-MRM Results Table’ in ‘Results Summary Tables and Data Tables’^12^).

## Data Records

To address the need for high quality affinity reagents for RAS-related proteins, we generated a suite of monoclonal antibodies to multiple unmodified and phosphorylated peptides from 20 proteins and 27 phosphopeptides. The characterization data and reagents for the RAS antibody resource are distributed among 6 data records, organized by assay type. The files associated with each Data Record are available in the public repositories cited in each of the following sections.

### Data Record 1: Resource of antibody reagents

Data Record 1 is displayed in the ‘Antibody Summary Table’ in ‘Results Summary Tables and Data Tables’^12^ and contains a list of all validated antibodies, including the type of antibody (mouse or rabbit), target gene symbol, and immunogen peptide sequence. Links to information for each antibody are available via the CPTAC Antibody Portal (https://antibodies.cancer.gov/). The publicly-available portal contains all the characterization datasets referenced below including a link to the University of Iowa’s Developmental Studies Hybridoma Bank (http://dshb.biology.uiowa.edu/) for availability of the research antibodies.

To generate a resource of affinity reagents we attempted mouse monoclonal antibodies to 96 unmodified peptides and rabbit monoclonal antibodies to 25 phosphopeptides. In total there were 21 proteins covered in the combined target list. Following immunization with the target peptide, we used peptide-based immuno-MRM to screen for antibodies from the best performing subclones. Synthetic light peptides were spiked into a background digest matrix and isolated by overnight incubation with the antibodies. The response was measured by MRM mass spectrometry by measuring the ratio of light peptide to heavy synthetic peptide (spiked into the sample following the capture). A positive response was determined by a ratio greater than that measured in a negative control sample (i.e. captured with no antibody). Based on this screening, we successfully identified mouse mAbs to 64 unmodified peptides (67% of the original 96 unmodified peptide immunogens), and 19 phosphopeptides (76% of the original 25 phosphopeptide immunogens). Because antibodies from multiple subclones were purified for some targets, a total of 119 mAbs were produced and purified. Validation testing in the applications indicated in this study produced 104 mAbs (78 mouse mAbs and 26 rabbit mAbs) validated in at least one application. The 15 mAbs that were produced and failed to achieve validation status in this study fall under two categories: (1) they had insufficient sensitivity for endogenous detection, or (2) there is another antibody to the same peptide sequence that featured superior performance.

### Data Record 2: Western blotting

Western blot images are presented in ‘Western blot data’^12^, and a table summarizing the data is presented in the ‘Western Blot Results Summary Table’ in ‘Results Summary Tables and Data Tables’^12^. All available Western blot data are available via the CPTAC Antibody Portal (https://antibodies.cancer.gov/).

The utility of the monoclonal anti-peptide antibodies for Western blotting was assessed using a Wes capillary electrophoresis platform. The antibodies were tested in two stages. First, antibodies were tested for detection of purified recombinant proteins, and antibodies that tested positive against recombinant proteins were subsequently tested in lysates from the MCF10A-KRAS breast cancer epithelial or the BxPC-3 pancreatic adenocarcinoma cell line. Blots were deemed positive if a band at the expected molecular weight (given by the vendor for the recombinant proteins or based on the molecular weight reported in UniProt) was observed. Overall, 63 (53%) of the 119 antibodies were positive against recombinant proteins using Western blotting. Of the 63 positives, 41 were also successful in detecting endogenous protein (for 17 proteins) in the cell lysates that were tested (see the ‘Western Blot Results Summary Table’ in ‘Results Summary Tables and Data Tables’^12^).

### Data Record 3: Immunoprecipitation

The ‘IP-MS Results Summary Table’ in ‘Results Summary Tables and Data Tables’^12^ lists the antibodies that were tested in the immunoprecipitation experiments, and the ‘IP-MS Peptide and Protein Intensity Table’ in ‘Results Summary Tables and Data Tables’^12^ lists all the peptide sequences and proteins that were identified in the IP-MS experiments. The mass spectrometry proteomics data have been deposited to the ProteomeXchange Consortium via the PRIDE^7^ partner repository with the dataset identifier PXD012130^8^.

61 of the antibodies that were positive in Western blotting using recombinant proteins were tested in immunoprecipitation-shotgun mass spectrometry. The experiments were performed using recombinant proteins, the MCF10A-KRAS cell line, and an MCF10A-empty-vector cell line. The antibodies were grouped into nine multiplexed groups and used to capture target proteins from a pool of recombinant proteins. Positive immunoprecipitation was determined by detection of peptides to the target protein in the LC-MS/MS analysis of the pull-downs. In total, 56 mAbs successfully captured recombinant protein. The positive mAbs were applied to immunoprecipitation of protein from cell lysates and analyzed by LC-MS/MS. We found positive detection of endogenous peptides from seven target proteins for 15 mAbs.

### Data record 4: Protein array

The ‘Protein Array Results Summary Table’ in ‘Results Summary Tables and Data Tables’^12^ summarizes the protein array analyses for each antibody tested, and the ‘Protein Array Raw Data Table’ and the ‘Protein Array Normalized Data Table’ in ‘Results Summary Tables and Data Tables’^12^ show the non-normalized and normalized electrochemical luminescence values for each positive antibody, respectively. The ‘Protein array data’^12^ file illustrates the normalized data as bar graphs. Results for Data Record 4 are available via the CPTAC Antibody Portal (https://antibodies.cancer.gov/).

The antibodies were characterized for application in protein array analyses using 62 of the Western-blot-positive mAbs. For these experiments, and for the immunohistochemistry experiments below, purified mAbs from recombinant clones of the hybridomas were used, since these purified mAbs showed less background binding than the purified mAbs that were produced from the hybridomas (data not shown). Cell lysates from the NCI-60 cell line collection and the MCF10A-KRAS cell line were dried on plates in a well-based reverse phase protein array format. Antibodies were added to the wells and allowed to incubate overnight, and binding signals were detected using tagged anti-mouse or anti-rabbit antibodies. Detection of a protein in a cell line was deemed positive, positive (weak), or negative if the normalized value was above 1.5, between 0.5 and 1.5, or below 0.5, respectively, indicated by green, grey, and red bars in the bar graphs in ‘Protein array data’^12^. Overall, 17 mAbs were positive in the NCI-60 cell lines and 16 mAbs were positive in the MCF10A-KRAS cell line (to 11 proteins).

### Data record 5: Immunohistochemistry

The ‘Immunohistochemistry Results Summary Table’ in ‘Results Summary Tables and Data Tables’^12^ summarizes the immunohistochemistry analyses for each antibody tested, and the ‘Tissue immunohistochemistry data’^12^ file displays the positive tissue immunohistochemistry results. All of the NCI-60 cell line and tissue IHC data are available on the Antibody Portal (https://antibodies.cancer.gov/).

The antibodies were evaluated for immunohistochemistry using the NCI-60 cell lines and ovarian, colon, lung, and breast cancer tissue samples. A total of 54 mAbs (eight mAbs that were negative by the protein array analyses were not included in the IHC analyses) were tested and evaluated for positive staining. In summary, 27 of the 54 mAbs were positive in these experiments (covering 13 proteins).

### Data Record 6: Peptide immunoaffinity enrichment and mass spectrometry (immuno-MRM)

The list of antibodies with successful detection of endogenous peptides in the cell line lysates tested is available in the ‘Immuno-MRM Results Table’ in ‘Results Summary Tables and Data Tables’^12^. Mass spectrometry data for Data Record 6 can be found at the Panorama Public database resource^6^ and ProteomeXchange^10^.

The ability for the monoclonal antibodies to detect endogenous analyte peptides from cell line lysate tryptic digests was evaluated using immuno-MRM. A combination of different conditions was employed to characterize the endogenous detection of analytes by immuno-MRM depending on the availability of reagents and timing of the experiments. Generally, between one and seven cell lines were tested per mAb, from a panel of nine cell lines (see Methods above) that were chosen based on literature and public database searches. Antibodies were tested individually or grouped into multiplexed groups and used to enrich the target peptides from digested lysates. The enriched peptides were analyzed by targeted MRM and positives determined by successful detection of the target peptide. Where there was more than one antibody to a given peptide sequence, we used the antibody with highest response during screening experiments. We found that 89 (86%) of the 104 mAbs (to 20 proteins) tested were positive for endogenous detection in the cell lysates. 19 (70%) of the 27 phosphopeptides and 57 (83%) of the 69 unmodified peptides were detected by endogenous immuno-MRM.

The high specificity afforded by the mass spectrometer provides several advantages in using peptide immuno-MRM as an antibody validation technique. The mass spectrometer detects the analyte peptide with high specificity and can distinguish between multiple peptides enriched by the antibody. For this reason, it is advantageous to obtain antibodies capable of detection of multiple forms of the peptide. For example, when targeting phosphopeptides, there are two types of mAbs obtained: i) mAbs that specifically capture only the phosphopeptides (termed “specific” mAbs), and ii) mAbs that capture both the phospho and unmodified peptide versions (termed “total” mAbs). In this dataset, there are some mAbs that can capture multiple different phosphorylated forms of a peptide, and even capture peptides from similar proteins with slightly different peptide sequences. For example, mAb CPTC-MAPK1-1, generated to the doubly-phosphorylated peptide VADPDHDHTGFL(pT)E(pY)VATR (pT185/pY187) of MAPK1, is able to capture eight distinct peptides: the doubly-phosphorylated peptide, both singly-phosphorylated peptides, the unmodified peptide, and the analogous 4 forms of the MAPK3 peptide (IADPEHDHTGFL(pT)E(pY)VATR (pT202/pY204)). For this dataset, mAbs positive by immuno-MRM were split between specific and total antibodies (~50% each). Furthermore, there are 19 mouse mAbs that were immunized using unmodified peptides that can capture both the unmodified sequence and the corresponding phosphopeptides (based on synthetic peptide testing). The ‘Immuno-MRM Results Table’ in ‘Results Summary Tables and Data Tables’^12^ lists all peptides that are captured by each mAb, as determined from screening studies.

## Technical Validation

Antibody validation was conducted under ‘consensus principles’^4^ describing best practices for validating the specificity and sensitivity of the antibodies in the intended applications. By testing multiple applications, orthogonal approaches can be used to validate the results obtained in any given application. In Western blotting, recombinant proteins and cell line lysates were used to validate the reactivity. Orthogonal testing (immunoprecipitation and immuno-MRM) confirmed the reactivities and validated the specificities of the mAbs (when possible). The mass spectrometry identifications in immunoprecipitation provide a measure of the specificity at the protein level for those antibodies with positive IP results. In addition, the IP-MS results provide a dataset to potentially identify unintended (off-target) direct interactions of the antibodies^21^. Differences in the numbers of antibodies positive in IP-MS vs. immuno-MRM arise primarily from two factors: (i) capturing proteins (IP-MS) vs. linear peptides (immuno-MRM), and (ii) antibodies in IP-MS were screened in a subset of the conditions tested in immuno-MRM (two vs. nine cell lines/treatments). Endogenous detection was used to validate the sensitivity for all applications. In immunohistochemistry, specificity and sensitivity were tested using endogenous signal in tissue microarray and a negative control (below). The results are limited to the conditions used for testing in this study. Of note, 13 of the 15 mAbs that were positive for endogenous detection in cell lines by IP-MS were also positive by endogenous immuno-MRM. Furthermore, 17 mAbs that were positive in the NCI-60 protein array experiments were also positive by NCI-60 immunohistochemistry testing. 4 mAbs (CPTC-AKT3-2, CPTC-FOS-1, CPTC-MTOR-3, and CPTC-RAF1-3) were validated in all assays that tested endogenous matrices.

Experimental controls were used to validate the datasets, as follows. Cell line identities were confirmed by DNA fingerprinting using STR (Short Tandem Repeats) CODIS (Combined DNA Index System) typing. For the MRM mAb screening, a negative control capture was performed using the same beads and peptide standard mixes or cell line digests without any mAb in the capture mix. Specificity of all MRM runs was assured using the light and heavy peptide chromatographic peaks to check matching retention times and matching relative transition areas. For the immunoprecipitation-shotgun mass spectrometry experiments, negative control captures were performed by performing a capture in the same manner as test conditions with no mAb present. To validate the signal intensities for the well-based reverse-phase protein array, each screening plate contained a PBST-only negative control and an MCF10A-KRAS positive control. Expression signals were collected from each cell line of the NCI-60 panel, and then an average gene expression signal from 60 cancer cell lines was calculated. The correlation of each gene detected by protein array was then examined against the positive MCF10A-KRAS cell line. For the IHC analyses, negative control samples were processed without primary antibody present.

## Usage Notes

All mAbs were produced within the CPTAC program and all reagents and data are available on the CPTAC Antibody Portal (https://antibodies.cancer.gov), via links to the nonprofit Developmental Studies Hybridoma Bank (DSHB) at the University of Iowa. These reagents are available for Research Use Only according to the DSHB terms of use agreement which can be downloaded at: http://dshb.biology.uiowa.edu/Order-forms. The mAbs are listed at the DSHB website by their CPTAC IDs, which are given in the ‘Antibody Summary Table’ in ‘Results Summary Tables and Data Tables’^12^.

Raw mass spectrometry data for the immunoprecipitation experiments are available for download in the raw format from PRIDE^8^. Specifically, on the project page for these experiments (project # PXD012130), readers can click on “Download Project Files” to arrive at a page where the raw files are accessible. In addition, detailed search results files and peak results files are also available on the same page. Last, at the bottom of the page is an Excel file that provides a key between the raw file names and the sample run identifiers (see the column headers in the ‘IP-MS Peptide and Protein Intensity Table’ in ‘Results Summary Tables and Data Tables’^12^).

MRM mass spectrometry data available on the Panorama repository can be downloaded in archived (i.e. ‘zipped’) Skyline^14^ documents, containing all relevant files. Following download, the documents can be extracted and opened using the Skyline platform available at https://skyline.ms/project/home/software/Skyline/begin.view. Extensive documentation and tutorials are also available on the same website.

## ISA-Tab metadata file

Download metadata file
